# Metarules, Judgment and the Algorithmic Future of Financial Regulation in the UK

**DOI:** 10.1093/ojls/gqad014

**Published:** 2023-08-20

**Authors:** Andromachi Georgosouli

**Keywords:** financial regulation, rules, digitalisation, algorithmic decision-making, legitimacy, governance, code

## Abstract

UK financial regulators are experimenting with the conversion of rulebook content into machine-readable and executable code. A major driver of these initiatives is the belief that the use of algorithms will eliminate the need for human interpretation as a deliberative process, and that this would be a welcome development because it will improve effectiveness while cutting time and costs for regulators and the industry alike. In this article, I set out to explain why human interpretation should be preserved and further harnessed if data-driven governance is to work at all. To support my thesis, I bring attention to the limited translatability of rulebook content into code, and to the difficulties for machines to engage with the full spectrum of tasks of analogical reasoning. I further contend that it would be desirable to preserve human interpretation on procedural grounds pertaining to the legitimacy of financial regulators. I conclude with recommendations about the future design of the financial rulebooks.

## 1. Introduction

In response to calls for a more data-driven approach to regulation,^1^ the Financial Conduct Authority (FCA) and the Bank of England are experimenting with the conversion of rulebook content into machine-readable and executable code.^2^ These efforts focus primarily on information gathering, but their implications reach far and are expected to affect all areas and aspects of regulation.^3^ The view of human interpretation as an impediment to the effectiveness of regulation is a key premise of the growing appetite for the rewriting of rules into code. This is evident in the statement of objectives of the regulators’ flagship pilot programme of Digital Regulatory Reporting (DRR).^4^ Chief amongst them is ‘to make reporting rules and instructions less reliant on human interpretation and implementation, and so improve the quality of regulatory data’.^5^ The view of human interpretation as a hurdle is also highlighted in a recent Bank of England Discussion Paper, which notes how vague regulatory instructions ‘can lead to “pain points” for firms in interpreting instructions’, causing delays and quality issues for the Bank.^6^

While experimentation is still ongoing, two schools of thought have emerged as regards the future place of human interpretation in regulation: on the one hand, those in favour of full automation and of ‘taking humans out from [a] large part of the solution development and interpretation phase’; and on the other hand, those who take a more cautious approach.^7^ In this article, I do not aim to argue against digitalisation. Rather, my aim is to explain why, at least from the legal point of view, we have good reasons to ensure that human interpretation remains an indispensable component of data-driven governance.^8^ Accordingly, the real challenge is not to find ways to eradicate the process of interpretation, but to embed and harness it as a practice. At a minimum, this requires designing rulebooks which will help their human users take advantage of their own general intelligence and of the specialist intelligence of machines as they go about ascribing meaning to rulebook content against an evolving ecology of commercial practice.^9^ I offer three arguments in support of my thesis. The first concerns the limited translatability of regulatory content into algorithms. The second draws attention to the finite capabilities of machines in making determinations about the kind of action that is required (eg with regard to the sort of data that needs to be reported) given the existing and foreseeable development of the relevant technology. The third brings attention to the participatory, deliberative and constructive character of human interpretation as a process, and contends that we would have reasons to preserve it even if it were possible to overcome the limitations discussed under the first two arguments.^10^

I develop my thesis on the following assumptions. Machine-readable and executable regulation consists of *metarules*, namely authoritative micro-directives which are expressed in algorithmic language and specify concrete courses of action (or omission, but I will leave this aside) for rulebook users, while enabling the execution of at least some aspects of that action by machines.^11^ I use the prefix ‘meta’ to convey the supervenience of code on regulatory content. I use the term ‘rule’ to mark the normative character of that content. I further assume that metarules are the outputs of algorithmic decision making, which bears the following two features: (i) functional autonomy in the performance of certain tasks (eg retrieval of specific data); and (ii) quasi-decisional autonomy, ie reliance on machine learning for the processing of inputs and the determination of outputs in a manner partially independent of human designers and operators, so that it remains compatible with the requirements of the Data Protection Act 2018 where relevant.^12^ Such outputs may be neither entirely predictable nor susceptible to reasoned explanation and justification, though this is consistent with humans retaining the capacity to intervene, eg to validate machine outputs, and the formal discretion to accept or reject them.

Following this introduction, I start my analysis by providing a brief account of the engineering of metarules as a response to the problem of legal uncertainty, leaving the discussion of other potential benefits of digitalisation and automation aside for another occasion (section 2). I present my three arguments in sections 3–5. In section 6, I conclude with a set of principles for the future design of financial rulebooks which are animated by the idea that *no stakeholder should become worse off* as a result of the use of metarules and automation.

In the interests of clarity and scope, I will focus on metarules derived from the binding content of the rulebooks of the FCA and the Prudential Regulation Authority (PRA), namely regulatory provisions earmarked here (as in the rulebooks) as ‘rules’, so that they are differentiated from non-binding guidance. My examples draw primarily on relatively detailed rules because they explicate the content of the high-level principles of the rulebooks and, as a result, they provide the natural starting point for exploring the conversion of rulebook content into metarules.^13^ The statutory objectives of the two regulators alongside the regulatory principles and the threshold conditions, uncodified common law principles, commercial practices and customs are also of relevance to the interpretation of regulatory law and add an extra layer of complexity, but their examination falls beyond the scope of my inquiry, and of most accounts of algorithmic regulation.

The impact of technology on the use of rules as instruments of social organisation and control has received growing attention in recent legal scholarship. By way of example, Aaron Wright and Primavera De Filippi have coined the term ‘lex cryptographia’ to describe the eventual rise of ‘rules administered through self-executing smart contracts’.^14^ In their turn, Anthony Casey and Anthony Niblett have declared the future ‘death of rules and standards’ thanks to machines translating complex legislative goals into ‘a vast catalog of simple commands for all possible scenarios’.^15^ The intersection of artificial intelligence, technology and the law in financial markets has also been researched extensively.^16^ The merits of a system of data-driven financial governance with little or no reliance on human interpretation has escaped systematic examination. My thesis seeks to address this gap in the literature.

## 2. The Engineering of Metarules as an Answer to the Problem of Legal Uncertainty

Financial regulators deploy a variety of rules to communicate their commands, expectations and guidance.^17^ In terms of their linguistic structure, these different types of rules can be seen as making up a spectrum of options, with highly specific rules (rules) standing at one end and vague rules (standards) standing at the other.^18^ Broadly stated rules tend to be durable and flexible, and they allow greater discretion in their interpretation. Detailed rules tend to provide greater certainty, clarity and predictability. Furthermore, their use seems to be more appropriate where the relationship between regulators and regulatees is one of mistrust. Neither economic analysis nor behavioural studies can provide a definite answer to the choice of legal form.^19^ However, they both corroborate the view that rules are more expensive than standards for regulators to make, while standards are usually more expensive than rules for regulatees to apply.^20^

Where the law is not sufficiently clear on a particular matter, regulatees need to spend time and resources to determine whether their behaviour complies with the law.^21^ Financial regulators try to reduce these costs pursuant to the regulatory principles of the Financial Services and Markets Act 2000 and specifically to that of proportionality, thus internalising part of the cost of legal uncertainty.^22^ When the regulated behaviour is frequent and homogeneous, (nearly) full information is assumed to be available *ex ante* and, as a result, detailed rules are not prohibitively costly to make.^23^ When the regulated behaviour is infrequent and heterogeneous, full information is not available at the point of rule making and vague rules (standards) are promulgated instead as a more affordable option when seen from the perspective of rule makers.^24^

Recent technological developments have the potential of cutting the costs of rule making significantly for the benefit of regulators and regulatees alike.^25^ Specifically, predictive, data storage and communication technology promises to improve the ability of financial regulators to collect and store information, make projections, design finely calibrated rules in algorithmic language, update the content of those rules and communicate them in (almost) real time.^26^ In short, it pledges to unlock the mutation of the existing financial rulebooks into a massive catalogue of metarules for the provision of constantly updated, context-specific, granular instructions to rulebook users.^27^

To see how this new type of financial rulebook might work, consider the use of traffic lights for the regulation of the flow of traffic:^28^ in a world without traffic lights, drivers would have to consult timetables and directives with prescribed intervals of stopping. With traffic lights, all these complexities are translated into a simple instruction: a red light or a green light, depending on whether drivers are required to stop or to continue driving. This is the simplest function of traffic lights, but more sophisticated functions are also possible. For example, some traffic lights adjust the duration of intervals or give priority to an ambulance in an emergency thanks to sensors which detect and predict the flow of the traffic in real time.

In a similar way to how traffic lights produce red and green signals to regulate traffic on public roads, it is at least conceivable that machines could be trained to produce metarules for the regulation of financial markets. Powered by advanced data and predictive analytics, machines would receive data input, identify the relevant rulebook provision, create a metarule that is a simple tailor-made instruction (eg in relation to a reporting requirement) and then communicate that instruction in real time or even execute the action that is required automatically (eg retrieval and submission of a specific bit of data in compliance with a reporting requirement) on behalf of human rulebook users.^29^ Subject to further improvements, the same technology could be deployed for the automatic detection of violations of regulatory law and even the automation of enforcement:^30^ administrative fines could become immediately payable, or human operators may be automatically restrained from taking any further action.^31^ A machine could also be programmed to produce a compliance score as a metarule to warn, for instance, a mortgage advisor that a particular recommendation would be in breach of the suitability requirements of the FCA Rulebook and, if required, even proceed to log them out from their desktop computer automatically, or otherwise block the completion of the transaction.^32^

From the point of view of computer engineering, we have technology in place with the potential to support the massive production and execution of metarules.^33^ Machine learning is a type of artificial intelligence that enables machines not just to do specific tasks (eg to retrieve, transmit, submit or update a specific bit of information), but also to learn without being explicitly programmed.^34^ A key advantage of this technology is that it allows real-time analysis of vast volumes of information for the identification of unusual correlations, patterns and emerging risks, and for making predictions. Blockchain and other types of distributed ledger technology, quantum computing, the Internet of Things as well as the convergence of Big Data and Big Compute are expected to increase data storage, access and processing, all of which are crucial for the further advancement of the analytical capabilities of computers with machine-learning software.^35^ The combination of machine learning with natural language processing or other types of semantic technology could also enable machines to read and process legal content for the execution of reporting and other tasks.^36^ An advanced type of hybrid machine learning, which is of particular interest here, is that of deep learning. One of the intriguing features of this technology is that it uses so-called ‘thought vectors’ to deconstruct language with almost mathematic precision and to translate and simulate the usage of natural language.^37^

The growth of machine-readable and executable financial regulation remains an incremental work in progress.^38^ Still, the possibilities of technological innovation in the field are so enticing that, as noted in section 1 above, they are already calling into question the case for retaining human interpretation as an aspect of the emerging system of algorithmic governance. In the next three sections, I explain why this is concerning. As I argue, the preservation of human interpretation is not an option—something that we might as well do without—but, in fact, a necessity if the envisaged data-driven approach to financial regulation is to work at all.

## 3. The Limited Translatability of Regulatory Content into Algorithms

The financial rulebooks consist of a combination of high-level principles, detailed rules and non-binding guidance.^39^ Taking the form of the FCA Principles for Business (PRIN) and the PRA Fundamental Rules (FR),^40^ the high-level principles are standards in terms of their function. They encapsulate benchmarks against which regulatees are to be assessed for their professional conduct and their financial health and soundness.^41^ The remaining myriad of rules and guidance are more detailed statements of the high-level principles of the rulebooks and are to be interpreted in the light of those principles.^42^ Thanks to their open-ended language, high-level principles allow a considerable degree of interpretive discretion. In addition, they amplify and reinforce the meaning of the more detailed provisions of the rulebooks. While the prominence of high-level principles in the rulebooks testifies to the survival of elements of a principles-based approach to regulation, their earmarking of PRIN and FR as ‘rules’ in the rulebooks suggests two things: the abandonment of the rhetoric of principles-based regulation and that the choice of regulatory approach and the choice of rule type are not necessarily aligned.^43^

The creation of machine-readable and machine-executable regulation requires coding.^44^ If we want a machine to do something for us, we need to give it an algorithm, ie a single list of rules presented in the right order for the machine to follow. A set of algorithms make up a code, while a system of codes makes up a computer software—for instance, a computer software that supports machine learning. Broadly speaking, software developers have three options when they code legal text: they can code the text themselves; develop an algorithm that trains machines to do the coding and produce outputs accordingly; or opt for a combination of both.^45^ A system of algorithmic financial regulation could make use of these options and produce algorithmic micro-directives of at least two kinds: (i) metarules for the execution of tasks like the automatic submission of data for reporting purposes;^46^ and (ii) metarules communicating compliance scores on the basis of a statistical model to enable humans to pre-test a step in order to see if it complies or not while retaining the formal discretion to accept or reject them.^47^

The crafting of metarules presupposes the translatability of rulebook content into its algorithmic equivalent, but the conversion of legal rules into code is an extremely challenging task.^48^ The root cause of these difficulties can be traced back to the kind of intelligence that machines are equipped with. Compared to humans, machines come with specialised intelligence. They exceed human capacity at specific tasks, but their focus is narrow and domain-specific, and therefore of limited transferability across domains. Machines are pre-programmed to deliver a specific goal, namely the one that is encoded in their software. Accordingly, their sophistication is a function of how effective they are in producing outputs that attain the goal in question (eg submission of specific data).

The more access they have to data, the more capable the machines become in performing pre-programmed tasks.^49^ Nevertheless, data accessibility is not enough. Machines do not process all types of data equally well.^50^ To perform well, they need to be fed with highly structured data, ie standardised bits of information with an exhaustively defined meaning. This is not to say that machines cannot cope at all with more open-ended and less clearly pre-defined data. They can, but the less structured the data, the more their capabilities diminish. The machines’ reliance on data of the highly structured type sets an important obstacle to the conversion of rulebook content into code. It requires software developers to, first, break rulebook content down into granular instructions and then convert those instructions into algorithmic language so that machines can read them and perform certain tasks. Reaching the requisite degree of granularity is usually not a problem for computer engineers. The question is how to attain granularity without risking any loss of meaning along the way.

Consider, for instance, SUP 16.11.5.R of the FCA Handbook, which, like most of the rulebook provisions, lacks exhaustive precision. SUP 16.11.5.R specifies that

A sales data report must contain sales data in respect of the following products: (1) retail investments; (2) pure protection contracts; (3) regulated mortgage contracts (but not further advances); (4) home purchase plans; (5) home reversion plans; (6) regulated sale and rent back agreements; (7) high-cost-term credit; and (8) home credit loan agreements.

Even though it is possible to rephrase the content of this legal rule into more granular attributes such as ‘sale’, ‘data’, ‘products’, ‘retail’ or ‘investments’, each one of those attributes also needs to be translated into more precise metadata to enable algorithmic conversion.

Translating SUP 16.11.5.R into more granular instructions for coding purposes is not as straightforward as it seems. Perhaps, the best way to illustrate this point is by considering HLA Hart’s well-known example of a rule according to which ‘no vehicle may be taken intothe park’.^51^ Suppose that software developers are training a machine to identify a vehicle and that, for the purposes of that training, they define ‘vehicle’ to mean ‘passenger cars’. This still leaves unclear whether the definition of the word ‘vehicle’ should include a truck, a wheelchair or a pram. No account of the meaning of the word ‘vehicle’ can include everything that is a vehicle and exclude everything that is not a vehicle. The relationship between the various uses of the word ‘vehicle’ is, as Ludwig Wittgenstein famously noted, like the relationship between members of a family.^52^ A resemblance exists, but it is not possible to give this resemblance any rigid definition.

Compared to SUP 16.11.5.R, certain more technically detailed reporting requirements seem to be more susceptible to algorithmic conversion from the point of view of computer engineering. Take, for example, SUP 16, Annex 21R of the FCA Handbook of Rules and Guidance. This legal rule describes the content of sales data in relation to mortgage-reporting requirements known as PSD001.^53^ SUP 16, Annex 21R specifies in extreme detail the data-reporting fields that must be completed and provides further guidance.^54^ For instance, one data field concerns the reference number of the product provider, which comes with a six-digit code. Another concerns the reference number of the product sold, which also comes with a six-digit code. Finally, a further data field is about the provision of financial advice at the point of sale, which comes with code ‘Y = advised’ or ‘N = not advised’. A technical legal rule of the type of SUP 16, Annex 21R of the FCA Handbook is most probably so exhaustive of meaning that its conversion into code is the least challenging. That said, it should to be noted that even the most technically detailed rules are not meant to be used in isolation from other parts of the rulebooks. To apply properly, they must be read in conjunction with rules of high or medium linguistic vagueness, namely rules whose algorithmic conversion is problematic, if not impossible, *ex ante*.^55^

The fact that the meaning of legal rules is context-dependent to varying degrees points to a further difficulty.^56^ Even when it is possible to convert the semantic content of legal rules into algorithmic language, it is not possible to capture the perpetually changing context within which these rules are meant to apply.^57^ Modern regulatory law does not shy away from the fact that its constituent rules and principles are anticipatory in nature and that, as a result, they communicate information that will be in need of refinement and clarification after the point of their making. In fact, it addresses this problem of uncertainty in the following manner: it expects rule users to interpret the content of the rule in question in the light of their cultural, social and economic background.^58^ The deployment of rigid and inflexible language is by and large counter-productive for the regulation of complex and dynamically evolving issues.^59^ To be sure, the ambiguity of open-ended legal language gives rise to a multitude of interpretations, but the ensuing risk of arbitrariness is to be kept under control in the course of interpretation on condition that it embeds a series of mechanisms of checks and balances. These mechanisms are deliberative in nature, and they rely on the rulebook users’ reflection, reason-giving and constructive contestation for the detection of errors on how a rule is to be understood.^60^

Compared to modern regulatory law, the architecture of machine-executable regulation is conspicuously different. We know from legal practice that what a rule means is not given *ex ante*. It is context-sensitive, and requires argument and contestation. Machine-executable regulation assumes the opposite. The mathematical compression of information into code makes information inaccessible (at least temporarily) while under the control of its *owner*, which is here understood in the broadest sense. The compressed information is presumed to have the following characteristics: it is uniform over the course of time, and independent from the person to whom the information is addressed and from other pieces of information.^61^

While the mathematical compression of information enables the transmission of bits of data with speed, integrity and confidentiality, the ensuing (albeit presumably temporary) collapse of space between the transmitted bit of information and its interpretation is deeply problematic from the legal point of view. This becomes clear once we think that something that is information to you is not information to me, and what is relevant or useful information to you may not be relevant or useful information to me.^62^ Training programmes for software developers and other professionals involved in coding and data validation, and the development of codes of best practice for the regulation of their professional conduct are two ways in which this concerning implication of the mathematical compression of information may be addressed.^63^ These measures ensure that machine outputs are checked and validated by humans with the necessary expertise. They can also help human rulebook users understand how machines process data, and what assumptions are embedded into their statistical modelling in order to be able to work out the grounds behind a metarule as a machine output. However, these measures do not restore the lost public space that regulatory law affords for interpretation to flourish as a practice of constructive deliberation through which the meaning of rules is brought into life. They are not meant to change the architecture of machine-readable and -executable regulation; they work within that architecture.

To conclude, the limited translatability of the content of the financial rulebooks makes plain that human decision making will be indispensable for the interpretation of regulatory content given the present and foreseeable stage of development of the relevant technology. On the one hand, it will be necessary for the application of those parts of the financial rulebooks that are not fully amenable to coding. On the other hand, it will be essential for addressing errors in relation to the fraction of rulebook content that is possible to translate into algorithmic language. For example, it will be required for factoring in issues that were not thought of at the time of the making of the computer program that generated the metarule in question, and for identifying any mistakes in the decisions that were informed by a faulty metarule.^64^

## 4. Specialist Intelligence and Its Limitations in Interpreting Regulatory Content

The interpretation of a legal text involves analogical reasoning.^65^ In its simplest form, this type of reasoning can be broken down into the following tasks: once a rule user has all the available information about the factual background of the issue at hand, they look at the history of the applications of the relevant law to figure out how that law may apply in the present situation.^66^ Specifically, they locate similar past situations and their determinations, they compare them with the current case, and they identify similarities and differences to discern the most relevant ones and form a conclusion about what the law requires in the current situation. In performing all these functions, a rule user does not just try to discover what others thought about a similar situation in the past.^67^ To do the job properly, they need to take a reflective stance. This involves coming up with a principle that makes the best sense of the relevant past decisions or determinations, and then applying that principle to the case at hand.^68^

In interpreting a legal rule, humans are able to grasp the meaning of that rule because they have a shared understanding of what a rule is, what following a rule is and what words mean.^69^ They attribute meaning in the light of the context within which the rule in question applies. Furthermore, they scrutinise and review each other’s attributions as active participants in a community of interpreters.^70^ Machines do not grasp the meaning of what they read the same way as humans do. A key feature of machine learning is that it is driven by a statistical model with a system of scoring which typically involves impenetrably complex calculations.^71^ The statistical model serves a specific goal in relation to which machines learn to mine data from vast datasets, identify correlations and patterns, infer information, make predictions and produce outputs.^72^ This goal may address a legitimate concern, for example, that of the consistent submission of specific data; but from that, it does not follow that it fully captures the policy objectives of financial regulators, or that it indeed yields correct outputs when assessed from the perspective of regulatory law.

Think of a computer software which trains machines to help regulatees comply with their legal obligation to share information and cooperate openly with the financial regulators as set out in PRIN 2.1.1 R of the FCA Rulebook and the identical FR 2.7 of the PRA Rulebook, respectively. To pre-empt the disclosure of all available information at all times and in all circumstances, suppose also that the computer software of my example trains machines to generate metarules to the effect of recommending non-disclosure each time the statistical model predicts that financial regulators are unlikely to follow up and, as a result, detect the non-release of pertinent information. Being informed by an irrelevant consideration—that of the likelihood of being caught withholding information—metarules of that kind would almost certainly lead to wrong regulatory outcomes. And, if I am right with this observation, then it is almost certain that these metarules would serve neither the policy objectives of financial regulation nor the delivery of strategic goals like the avoidance of a culture of creative compliance, amongst others.

Suppose now that a machine is trained to generate metarules to help regulatees comply with a wide range of FCA Rulebook provisions that mandate them to act with honesty when dealing with their clients.^73^ To differentiate between truthful and deceptive statements and produce a metarule accordingly, the machine of my example analyses digital records of client communications for linguistic markers of deception and then generates a compliance score to a given set of statements. The software does not capture the essence of dishonesty. Instead, the embedded statistical model works with indirect (and not always transparent) clues of dishonest communication to predict whether a particular communication will be earmarked as honest or not.^74^ Examples of indirect clues might be the frequency of avoiding first person singular pronouns, and the ratio of negation, equivocations and other linguistic patterns.^75^ Predictions of that sort are grounded on certain assumptions—for example, the idea that there is a causal interface between one’s language and cognition, and that telling a lie is more cognitively taxing then telling the truth. That notwithstanding, the reliability of these predictions is questionable partly because the statistical model ignores a range of other factors that are known to affect linguistic patterns—notably, underlying medical conditions and background noise.

Computer-based textual analysis is also blind of context. To address this issue at least in part, software developers often introduce an element of randomness to improve the learning capabilities of machines. For instance, they design software that programmes machines to gather additional data, learn from it and adjust their ‘thinking’ accordingly, each time the machines detect linguistic patterns that are not pre-programmed to identify as dishonest. While randomness improves the accuracy of machine outputs and helps pre-empt ‘gaming’—namely, strategic behaviour that aims to abuse the system—it concurrently increases opacity.^76^ Undoubtedly, computer engineers can be called upon to explain how the machine produces a certain output, and therefore their role in restoring a degree of transparency is crucial. That said, they have neither the legal training nor, indeed, the *de jure* power to clarify the circumstances under which a certain kind of conduct would amount to dishonesty in the eyes of the law.

This is not to say that human recommendations are always fully transparent. Think, for instance, the provision of legal advice.^77^ Clients often defer to the recommendations of their solicitor without always requiring a detailed account of all the factors that informed their legal advice. However, from that, it does not follow that their legal advice is unjustifiable or unexplainable. The solicitor operates within established frameworks of professional competence, independence and accountability, and they are prepared—when required—to explain and justify their recommendations. Like the solicitor of my example, financial regulators and other public decision makers operate within established frameworks of competence, independence and accountability through various mechanisms of appeal and scrutiny. Their decisions are reviewable, explainable and justifiable.

Finally, a further point of difference between human interpretation and machine-outputted determinations of regulatory content is that, in the latter case, the formation of an underlying structure of meaning is absent. The development of a semantic web is, of course, possible, but machine-learning technology does not require an initial concept of a pre-programmed rule structure.^78^ While this is an important advantage in one sense, it is troubling in another. Precisely because it does not presuppose the modelling of a semantic web, this type of technology exhibits greater scalability. However, the internal logic of machine learning does not follow established rules of inference, as humans do when they engage in interpretation; consequently, it cannot guarantee interpretive coherence.^79^ What it does is conduct statistical analysis and, accordingly, generate micro-directives with the sole criterion of the delivery of the automated system’s goal. The generated metarules may be connected with the specific goal that the automated system is designed to serve, but they are not connected to each other.^80^

To conclude, machines can carry out a series of tasks of analogical reasoning. They can retrieve factual information, identify matching past legal facts, enlist their similarities and differences, rank them in terms of relevance and use statistical modelling to output compliance scores at great speed. What they cannot do—at least, not yet—is to root interpretive determinations on judgments of principle according to public criteria that are open to intelligible scrutiny and contestation. Humans do better in performing this task of normative reasoning because of their moral imagination and capacity of critical judgment, but they are slow in navigating through voluminous legislation and case law for the retrieval of factual information or the identification of matching past legal facts without the aid of machines. To be sure, humans make mistakes, but at least we have a fairly comprehensive understanding of the nature of human error, and we can anticipate it. For example, judicial review offers a robust, albeit imperfect, pathway for the contestation of mistaken decisions in public governance, while supervisory visits and investigations help expose and scrutinise errors made by the regulatees.^81^ By contrast, our understanding of machine error is rudimentary at present and, as a result, our tools to respond to it are lacking in sophistication.

## 5. A Proceduralist Justification for the Preservation of Human Interpretation

Were we to overcome the limitations discussed under the first two arguments and, as a result, able to train machines to produce the perfect metarules, would we still have reason to preserve human interpretation? If regulatory law were there only to communicate to regulatees what they may or may not do, we would most probably be better off just by switching to metarules. However, the picture is more complex. In the UK, as in other modern democracies, it seems to be the case that regulatory law does more than that. It harbours an interpretive practice of constructive deliberation that cuts across decisions on the making and application of rules despite the procedural differences between the two processes.^82^ Accordingly, a question to ask is whether, absent that interpretive practice, an algorithmic scheme of financial markets governance would be legitimate.^83^

At this juncture, it is important to note that the question of legitimacy is not confined to the interests of the members of the regulated financial industry only. It also concerns consumers and virtually everyone affected by the decisions and actions of financial regulators. That said, in the remainder of this section, I shall construe financial regulation as a dyadic relationship between regulators and the regulated industry as a matter of priority and on the grounds of simplicity. My thinking is the following: if I can show that my procedural justification applies to the relationship between regulators and regulatees, then further questions can and should be asked on whether insights of my analysis could apply to financial regulation as a polycentric regime involving multiple stakeholders.

Focusing on the relationship between regulators and the regulated industry, it is clear that part of the financial industry demands digitalisation.^84^ Furthermore, it may even appear to be prepared to part with the opportunity to have a voice on how specific rulebook content is to be understood in supervisory visits or other interactions with regulators. From this, it does not follow that algorithmic financial regulation would be legitimate if human interpretation were to be progressively eradicated as an aspect of regulatory law, for two main reasons. The first is that advocates of digitalisation and automation form an interest group that is not necessarily representative of the entire financial industry or, indeed, of other stakeholders. The second reason concerns the nature of the question I am posing, which is a question of principle and not one of empirical fact.

With these observations in mind, one might be tempted to follow a different route in the attempt to answer the question of legitimacy in the affirmative. This time, they may appeal to the regulators’ superior expertise to provide a solution to a coordination problem—all courtesy of technological advancement.^85^ Specifically, they might say that, if regulatees would do better—for instance, in complying with reporting rules or in meeting capital adequacy requirements, by following metarules than by working out what to do on their own—then the authority of financial regulators would be legitimate. Despite its plausibility, this *substantive* test is flawed, because it fails to account for the procedural dimension of legitimacy in public governance.^86^

Several practices are structured by roles that have the attribute of authority in the sense that participation in those practices generates relationships that involve a right to rule for certain members and an obligation to obey for certain others.^87^ The relationship between teachers and their students is a notable example.^88^ Suppose that I am receiving lessons from a piano tutor in the hope that one day I will become a virtuoso of Arnold Schoenberg’s compositions. It is fair to say that I am more likely to succeed if I follow their instructions. My piano tutor has a claim that I follow their instructions because of their superior technique, specialist knowledge and expertise.^89^ Crucially, the justification of their authority over me does not turn on how they decide what is best for my tuition. As long as I am progressing well, it makes little difference to me, for example, if my tutor reads all the relevant literature on the 12-tone compositional structure of Schoenberg’s pieces or instead consults someone else on this issue in preparation for my piano lessons.

Even though the same could be said for the authority of doctors *vis-à-vis* their patients and a multitude of others whom we regard as experts in their field, the nature of the authority of financial regulators is different. We care a lot about *how* financial regulators reach decisions, which they will then communicate as orders, instructions or commands, or *how* they see to their proper application.^90^ Even though regulators are not required to obtain the consent of the regulatees to make, apply or even enforce the rules they make, we expect the regulators’ decisions to be the outcome of a participatory and inclusive process of constructive deliberation, and that those at the receiving end of commands should have a degree of (or at least an opportunity for) engagement in this process. These procedural aspects matter to us for several reasons. Chief amongst them is our commitment to principles of equality and personal autonomy.^91^ While the former commands that regulatees are involved in the making of decisions that are highly consequential to them, the latter requires that they are empowered to take control of their own affairs and projects. We also believe that, as moral agents and rational beings, regulatees should be allowed the space for exercising judgment and discretion when circumstances so demand. A participatory process is also valuable to us because rigorous debates help us reach better decisions and because it promotes industry enrolment, which is crucial for the more effective delivery of public policy objectives.^92^

These observations make plain that, unlike scientific experts, the authority of financial regulation is *practical* and not *epistemic* in nature.^93^ An implication of this is that the obligation of regulatees to obey is grounded on the presence of a participatory process of constructive deliberation that we regard as worthy of the regulatees’ acceptance and, as a result, legitimate, because it treats them with equal concern and respect or because it is seen as instrumental to the delivery of an intrinsic good, such as that of a shared interest in being able to autonomously control one’s projects. Accordingly, the criterion for assessing legitimacy is first and foremost *procedural* and not substantive in nature. The fact that the legitimacy of financial regulators can hardly ever be reducible to claims about their technocratic expertise and effectiveness is further verified by the range of attributes that we identify as indispensable to any form of public governance with a credible claim to legitimacy.^94^ This is not to say that expert input and effectiveness are not essential; rather, it is to make the point that a legitimacy test that focuses only on the regulators’ superior expertise and capabilities is liable to miss out other important considerations, namely our expectation that decisions ought to be the outcome of a dialectic process that allows space for input and contestation when rules are made as well as when they are applied.^95^

It is difficult to see how the legitimacy of the regulators’ authority could be established if human interpretation were to be overtaken by algorithmic decision making and automation. Consider, for instance, liquidity reporting requirements—a highly technical cluster of rules, which the Bank of England is seeking to convert into code.^96^ The fact that they involve metrics and complicated calculations does not make these rules less consequential for regulatees. They feed into reports about the financial health and soundness of the regulated firms with micro-prudential and macro-prudential implications. Regulatees have every reason to want to have some control over their liquidity data and how it is used to ensure that it is correctly interpreted and that the conclusions to be drawn are valid.^97^ None of this is possible without space for a dialectic interaction with the competent financial regulator—typically, during supervision or during investigations when the regulatees’ compliance is called into question. Undoubtedly, the task of interpretation is burdensome, and humans may exhibit predictable and often irrational behaviour,^98^ but the point here is that they remain moral agents capable of self-reflection and of taking responsibility for their acts and omissions.^99^

These virtues of human discretion, judgment and agency are deeply embedded in the UK style of financial supervision. The supervisory approach of the FCA and the PRA is judgment driven.^100^*Prima facie* financial regulators exercise discretion over the interpretation of available factual evidence to assess things like the magnitude of emerging risks and the compliance of a specific type of behaviour with the financial rulebooks. In reality, the exercise of judgment is diffused. Following the long British tradition of self-regulation in financial markets governance, members of the industry are not passive recipients of regulatory commands.^101^ They are expected to exercise discretion as they engage in a process of self-reflection on how they ought to run their business. Regulatory interventions are on the menu to communicate findings and interrogate business culture, and require a particular course of action. However, these interventions are meant to treat regulatees not as mere objects to be controlled, but as subjects capable of ruling themselves and of being accountable for their actions. To sum up, at the heart of judgment-led supervision lies an enduring commitment to a participatory and constructive process of interpretation, and rightly so, because without it, the legitimacy of the authority of financial regulators would be wanting.

## 6. Designing the Rulebooks of the Future

In view of the difficulties discussed above, the idea of ostracising human interpretation from financial regulation should be abandoned. Instead, a more cautious approach is required, namely one which will embed human interpretation into the fabric of data-driven governance and seek to harness its deliberative and constructive elements by helping human rulebook users take advantage of their own general intelligence while also benefiting from the specialist intelligence of machines.^102^ So, if algorithmic decision making is to coexist with human decision making, how are we to design the financial rulebooks of the future?

A good starting point here is to decipher the nature of the relationship between the natural language that underpins human decision making and the algorithmic language that enables the execution of various tasks by machines. There is no doubt that the use of code promises to lift barriers, gaps and other obstacles that currently inhibit procedural efficiency. However, Frank Pasquale notes that:

While computer code and human language both enable forms of communication, the affordances offered by each are distinct and, in many respects, mutually exclusive. Code seeks to eliminate the forms of ambiguity and flexibility characteristic of much language, including legal language.^103^

In the first instance, if both forms of communication were to coexist, the tension would be inevitable.

To be sure, the tension between linguistic precision and vagueness is always present in financial regulatory law.^104^ The open-ended language of the statutory objectives of the FCA, the PRA and a good number of rulebook provisions coexists with the more precise formulation of a plethora of more detailed rules and non-binding guidance.^105^ However, the proliferation of metarules is set to push the trend towards greater linguistic granularity even further, as this will be necessary to promote standardisation, consistency and accuracy. If this trend is left unattended, it will progressively close down the necessary linguistic space for interpretive judgment. Moreover, the emerging data-driven architecture will place rulebook users under growing pressure to lean towards a particular outcome or towards a particular way in which a task is to be carried out.^106^ To understand how this might happen, suffice to consider the impact of automation on what might be described as the incentives problem in judgment-led supervision.

The fact that regulators and regulatees are allowed the necessary linguistic space to exercise judgment (at least for the time being) does not mean that they are always willing to exercise it. Cognitive limitations, heuristics, biases and ulterior motivations shape their attitudes.^107^ For members of the financial industry, the exercise of judgment is routinely associated with the risk of interpretive error, which they would rather avoid due to concomitant costs.^108^ For regulators, judgement-led supervision quite often implies greater exposure to blame in case of failure. Undoubtedly, financial regulators do (and should) have the power to overrule machines, but if their judgment is to be increasingly perceived as relying on *personal views* instead of the more *scientific* machine-outputted micro-directives, their future confidence in their judgment should not be taken for granted. For example, if at some point the bureaucratic culture within their internal organisation commands that an algorithmic prediction shall be followed as a matter of best practice, it will be difficult to insist on the value of judgment. Successful judicial review proceedings might challenge that emerging practice on the grounds of failure to consider relevant factors or due to improper delegation of power to an algorithm amongst others.^109^ However, the potency of administrative law to counteract this trend is not a reason for complacency. Looming pressure for the accommodation of emerging best practice also needs to be factored in because of its propensity to instigate a shift of paradigm with potential repercussions on future perceptions about appropriate industry attitudes to automation and concomitant business culture.^110^

With the propagation of metarules, the incentives problem may worsen due to the growth of automation bias and the concomitant phenomenon of de-skilling. Automation bias is the unfounded but nevertheless strong belief that, compared to humans, computers are more rational and objective in their decision making.^111^ De-skilling is intertwined with automation bias.^112^ Decisions about the use of legal rules involve normative reasoning skills, including the capacity to sense a degree of social connection, critical judgment, empathy and moral imagination.^113^ As rulebook users will have less opportunity to develop those skills, there is a risk that their ability to engage in normative reasoning and to appreciate the moral choices of their actions will atrophy.^114^ Furthermore, in the absence of any countermeasure, automation bias will show no sign of abating despite the fact that machines will continue to take faulty decisions by law’s standards.

To ensure that any problems are kept under control and that no stakeholder becomes worse off as a result of the increasing use of metarules in the future, it is necessary to establish the primacy of natural language for the communication of rulebook content. The following principles are therefore recommended for the design of the financial rulebooks: the first of those principles is the principle of *optimisation*. According to this principle, financial rulebooks should be drafted in a way that helps users benefit from both the general intelligence of humans and the specialist intelligence of machines. The remaining principles introduce qualifications to the principle of *optimisation* and are mutually reinforcing. Specifically, the principle of *user-centricity* requires that human rulebook users be treated as active members of the regulatory community rather than passive recipients of explanations about what they are to do or what they did wrong in a given situation.^115^ The implication of that principle is that human judgment takes precedence over machine outputs and that humans remain in control of and are ultimately responsible for machine determinations of regulatory content as befits their moral agency. Next is the principle of *mutual exclusivity* of natural language and algorithmic language as forms of communication. According to that principle, algorithms should not be introduced into value-laden assessments for, otherwise, there is a risk that judgment and discretion will be eroded. Finally, the fourth principle is that of *non-substitutability* of human decision making, which is here broadly conceived to underscore two things: the crucial function of human decision making for the detection of errors by law’s standards and the deliberative character of the process of interpretation that is embedded in the use of rules as a prerequisite of regulatory legitimacy. Taken together, these design principles are desiderata that are intended to serve as criteria for assessing how far to go with the coding of rulebook content.

To make sure that future rulebooks pass the test of all the above four design principles, their content must reflect a clear division of labour between human decision making and machine decision making. This can be done through the progressive introduction of two-tiered rulebook content, expressed in natural and algorithmic language respectively. The allocation of different rules to humans and machines would not be easy and will most probably require a radical reconceptualisation of the current content of the rulebooks. One possibility might be to split tasks between humans and machines in terms of those aspects of analogical reasoning that humans are known to do better compared to machines and vice versa, and to rewrite the content of the rulebooks accordingly.

Written in natural language and intended for humans, the first tier would be similar to the existing rulebooks. It would consist of legal rules of varying degrees of linguistic vagueness and precision to accommodate the use of general human intelligence. It would cover the full spectrum of rules and requirements of the existing financial rulebooks to regulate how human decision makers root interpretive determinations in judgments of principle and to enable human oversight over algorithmic decision making. The second tier would be for machines. It would be written in algorithmic language to facilitate the use of specialist machine intelligence for the execution of all other tasks of analogical reasoning under the necessary human oversight. Examples include the retrieval of factual information, the identification of the applicable set of rules, the navigation of the history of their past applications, the generation of compliance scores and the mapping of similarities, differences or other correlations.

The proposed financial rulebooks will be greater in size, volume and complexity than the existing ones and they will not come cheap.^116^ However, the point here is that, as long as they conform to the design principles described above, they should be able to ensure that the penetration of algorithms into the governance of financial markets will no longer pose a serious threat to all those skills that are necessary for human rule users in order to exercise judgment and discretion when circumstances so require. Ultimately, it will be left for regulators to explain, for instance, why a machine output should be followed, called into question or even overridden in the case at hand. On their part, regulatees will be able to reap procedural and cost-efficiency benefits, but they will still be required to exercise judgment on what is prudent, fair, honest and reasonable where appropriate.^117^ More generally, all determinations of regulatory content would continue to be open to public scrutiny, contestation and review by human rulebook users.

At present, technology solution vendors and other private sector technology firms are at the forefront of the conversion of regulatory instructions into code and of the authorship of the relevant protocols and operating manuals. The conspicuous presence of the private sector ensures that the latest cutting-edge research feeds into the development of regulatory technology, but there is a downside to this arrangement: to all intents and purposes, the coding of the more specific instructions and templates is left in the hands of a group of stakeholders that neither has the *de jure* power to clarify the content of regulatory law nor is subject to appropriate accountability requirements. To address this problem, it is further recommended—albeit not without controversy—that financial regulators take direct control over the algorithmic conversion of the rules they make, and that they continue to develop synergies with all relevant stakeholders to benefit from input. In this regard, the contribution of lawyers would be crucial in helping computer scientists appreciate the nature of modern regulatory law, the salience of constructive deliberation, and the need for an algorithmic architecture of financial regulation apt to accommodate human action, individual freedom and personal autonomy.^118^

To ensure that the imperatives of the proposed design principles will not be diluted in practice, it is also essential to be clear about the legal status of all those metarules that will make up the algorithmic tier of the financial rulebooks. Metarules share certain properties with legal rules, but they lack others. For example, like legal rules, metarules exhibit a sort of linguistic structure to the extent that code can be loosely described as a form of algorithmic language serving as the alphabet and grammar of a data-driven system of financial regulation. They also appear to display a normative dimension in the sense that they have the potential to exert legal effects in the form of pre-programmed interpretive guidance. Given these similarities, it might be tempting to think of metarules as *sui generis* soft law. Nevertheless, this idea should be resisted at least at present because their making does not correspond to the same procedural criteria that apply to the making of the PRA and FCA Rulebooks. The supremacy of legal rules over their algorithmic equivalent should therefore be acknowledged firmly and unequivocally in primary legislation so that it becomes mandatory to all. This option may lack the appeal of market-based voluntary arrangements, but it merits consideration because it guarantees that the primacy of legal rules will not hinge on the good will of rulebook users or on the effectiveness of contractual mechanisms of enforcement.^119^

## 7. Conclusion

In their attempt to implement a more data-driven approach to regulation, the FCA and the Bank of England are experimenting with the creation of rulebook content that can be read and executed by machines. The current plans to introduce a competitiveness and growth objective for the FCA and the PRA and to place digitalisation at the core of post-Brexit financial regulation hint that these efforts will be intensified in the years to come.^120^ Rewriting rulebook content into code promises to lift the burden of interpreting an increasingly complex volume of rules and requirements, with profound benefits for the industry and the regulators alike. At the same time, it raises important questions about the future place of human interpretation as a process of constructive deliberation and about how far to go with automation in algorithmic financial regulation.

As I have argued, we have good reasons to ensure that human interpretation remains an indispensable component of the emerging data-driven governance. To support my thesis, I brought attention to the limited translatability of regulatory content into algorithmic language, and to the difficulty of machines to engage with the full spectrum of tasks of interpretation as a form of analogical reasoning. I further showed that, even if it were possible to overcome these problems, it would be desirable to preserve human interpretation on procedural grounds pertaining to the legitimacy of the regulators’ authority, namely on grounds that go beyond considerations of efficiency and evidence-based expertise.

If human interpretation is to remain a feature of data-driven regulation, it is essential to have in place rulebooks that will help their human users take advantage of their general intelligence as well as the specialist intelligence of machines. In view of this, I concluded the discussion by putting forward a series of design principles for the drafting of the rulebooks of the future. These are the principles of optimisation, user-centricity, mutual exclusivity of natural language and algorithmic language, and non-substitutability of human decision making. The proposed rulebooks will not be perfect and their design will require further refinement. Nevertheless, their creation will be an important stepping stone in that it will ensure that determinations of regulatory content remain susceptible to public contestation and scrutiny by humans, so that no stakeholder becomes worse off as a result of the penetration of algorithms into the governance of financial markets.

My thesis allows for a more balanced assessment of the benefits of algorithmic financial regulation. It also sheds light on the often-neglected procedural aspects of human interpretation and what they stand for in public governance with wider implications on other fields of regulatory law and legal studies on regulation more generally. To be sure, my analysis does not have all the answers to the problems that we are likely to encounter as digitalisation will progress and possibly expand beyond the information gathering space that is currently being contemplated. However, it merits consideration because, at the very least, it shows that human interpretation is not an impediment but a prerequisite of regulatory effectiveness. It is therefore imperative that it is treated as such. The goal for the years to come, then, is to develop a digital architecture that, instead of negating, enhances the inclusive, participatory and constructive character of interpretation as a process while, at the same time, improves the aptitude of human rulebook users for judgment and discretion when circumstances so demand.

## Footnotes

1 Bank of England, ‘FCA and Bank of England Announce Proposals for Data Reforms across the UK Financial Sector’ (News Release, 7 January 2020) <www.bankofengland.co.uk/news/2020/january/fca-and-boe-announce-proposals-for-data-reforms-across-the-uk-financial-sector> accessed 20 August 2021.

2 FCA, ‘Data Strategy’ (first published 7 January 2020; last updated 6 August 2020) <www.fca.org.uk/publications/corporate-documents/data-strategy> accessed 20 August 2021; Bank of England and others, ‘Digital Regulatory Reporting: Phase 2 Viability Assessment’ 4, 8 and 17 <www.fca.org.uk/publication/discussion/digital-regulatory-reporting-pilot-phase-2-viability-assessment.pdf> accessed 20 August 21; Bank of England, ‘Transforming Data Collection from the UK Financial Sector’ (Discussion Paper, January 2020) (Bank of England Discussion Paper) paras 7.16–7.24 <www.bankofengland.co.uk/-/media/boe/files/paper/2020/transforming-data-collection-from-the-uk-financialsector.pdf?la=en&hash> accessed 25 August 21. =6E6132B4F7AF681CCB425B0171B4CF43D82E7779>.

3 FCA, ‘Digital Regulatory Reporting’ (first published 1 November 2017; last updated 14 October 2020) <www.fca.org.uk/innovation/regtech/digital-regulatory-reporting> accessed 20 August 2021; Bank of England, ‘Transforming Data Collection from the UK Financial Sector: A Plan for 2021 and Beyond’ (23 February 2021) (Bank of England Transforming Data Collection Plan). <www.bankofengland.co.uk/paper/2021/transforming-data-collection-from-the-uk-financial-sector-a-plan-for-2021-and-beyond> accessed 20 August 2021.

4 FCA, ‘Digital Regulatory Reporting’ (n 3).

5 FCA, ‘Pilot Phase 1 Report’ (June–December 2018) 7. <www.fca.org.uk/publication/discussion/digital-regulatory-reporting-pilot-phase-1-report.pdf> accessed 20 August 2021; see further Bank of England and others (n 2) 4 and 6 (identifying interpretive problems as major drivers of the high cost of industry compliance in the UK).

6 Bank of England Discussion Paper (n 2) 2.

7 Bank of England Transforming Data Collection Plan (n 3) para 4.2.

8 Bank of England Discussion Paper (n 2) para 7.16 (conceding, albeit tentatively, that interpretation will still be required).

9 Here I am following Margaret Boden, who describes the various types of artificial intelligence (AI) as ‘specialist systems’ of intelligence to distinguish them from the ‘general intelligence’ of humans. MA Boden, *Artificial Intelligence, A Very Short Introduction* (OUP 2018) 18.

10 A Georgosouli, ‘Regulatory Interpretation: Conversational or Constructive? (2010) 30 OJLS 361 (discussing the nature of interpretation in regulation).

11 AJ Casey and A Niblett, ‘Focus Feature: Artificial Intelligence, Big Data, and the Future of Law—Self-Driving Laws’ (2016) 66 UTLJ 429 (using the term ‘micro-directive’).

12 Whilst the Data Protection Act 2018 applies to personal data, the legislator was clearly concerned with automated decisions without human control. Of particular relevance here are ss 49(1) and 50. The former stipulates that ‘a controller [of personal data] may not take a significant decision based solely on automated processing unless that decision is required or authorised by law’. The latter adds that a data subject can request an automated decision to be reconsidered or a new decision to be made ‘not based solely on automated processing’. See further K Yeung, ‘Why Worry about Decision-Making by Machines?’ in M Lodge and K Yeung (eds), *Algorithmic Regulation* (OUP 2019) 21, 22–3.

13 This is discussed further in s 3 below.

14 A Wright and PP De Filippi, ‘Decentralized Blockchain Technology and the Rise of *Lex Cryptographia*’ (10 March 2015) <https://ssrn.com/abstract=2580664> accessed 20 August 2021.

15 AJ Casey and A Niblett, ‘The Death of Rules and Standards’ (2017) 92 Ind LJ 1401.

16 The scholarship focuses primarily on the development and legal implications of financial technology (FinTech), technology governance and competition law issues associated with sandboxes for FinTech experimentation. See E Micheler and A Whaley, ‘Regulatory Technology: Replacing Law with Computer Code’ (2020) 21 EBOR 349; S Omarova, ‘Technology v Technocracy: Fintech as a Regulatory Challenge’ (2020) 6 Jahresschrift fur Rechtspolitologie 75; E Avgouleas and A Kiayias, ‘The Promise of Blockchain Technology for Global Securities and Derivatives Markets: The New Financial Ecosystem and the “Holy Grail” of Systemic Risk Containment’ (2019) 20 EBOR 81; R Van Loo, ‘Making Innovation More Competitive: The Case of Fintech’ (2018) 65 UCLA L Rev 232; W-G Ringe and C Ruof, ‘A Regulatory Sandbox for Robo Advice’ (2018) EBI Working Paper Series 2018—No 26 <https://papers.ssrn.com/sol3/papers.cfm?abstract_id=3188828> accessed 17 June 2022; DW Arner, J Barberis and RP Buckley,’ FinTech, RegTech, and the Reconceptualization of Financial Regulation’ (2017) 37 Northwestern Journal of International Law & Business 371. In parallel to this literature, a more theoretically oriented branch of discourse examines the advent of algorithmic regulation, and the impact of technology on legal concepts and doctrines. See R Brownsword, E Scotford and K Yeung (eds), *The Oxford Handbook of Law, Regulation and Technology* (OUP 2017); M Lodge and K Yeung (n 12); M Hildebrandt, ‘Law as Information in the Era of Data-Driven Agency’ (2016) 79 MLR 1.

17 Financial Services and Markets Act 2000 (FSMA 2000) pt 9A (providing the legal basis of the rule-making powers of the FCA and the PRA). Classifications of different types of rules abound in the literature. See notably, F Schauer, *Playing by the Rules: A Philosophical Examination of Rule-Based Decision Making in Law and in Life* (Clarendon Press 1991) 1–16; R Baldwin, M Cave and M Lodge, *Understanding Regulation, Theory, Strategy and Practice* (OUP 2012) 230, 296–311; A Ogus, *Regulation, Legal Form and Economic Theory* (Hart Publishing 1994) 150–79; J Black, *Rules and Regulators* (Clarendon Press 1997) 7–10.

18 F Schauer, ‘The Convergence of Rules and Standards’ (2003) 3 New Zealand Law Review 303, 307 (with further reference to literature demonstrating the diversity of approaches taken on the distinction between rules and standards).

19 RB Korobkin, ‘Behavioral Analysis and Legal Form: Rules v Standards Revised’ (2000) 79 Or L Rev 23, 58; KM Clemont, ‘Rules, Standards and Such’ (2020) 68 Buff L Rev 751, 760. For a classic economic analysis of legal rule making, see I Ehrlich and RA Posner ‘An Economic Analysis of Legal Rule-Making’ [1974] JLS 257, 286 (identifying four types of costs: the cost of rule making, the cost of enforcement, the cost of compliance, which is defined as the cost imposed on the industry, and social costs understood as the costs imposed by regulatory offences). On the economic analysis of law making and the ‘rules versus standards’ debate, see further L Kaplow, ‘Rules versus Standards: An Economic Analysis’ (1992) 42 Duke LJ 557; F Schauer, ‘The Tyranny of Choice and the Rulification of Standards’ (2005) 14 Journal of Contemporary Legal Issues 803; CS Diver, ‘The Optimal Precision of Administrative Rules’ (1983) 93 Yale LJ 65, 73–4. On the contribution of behavioural economics to the traditional economic analysis of law, see notably C Jolls, C Sunstein and R Thaler, ‘A Behavioural Approach to Law and Economics’ (1998) 50 Stan L Rev 1471, 1474, 1545; E Zamir and D Teichman, *Behavioral Law and Economics* (OUP 2018) 1. For a classic criticism, see RA Posner, ‘Rational Choice, Behavioral Economics, and the Law’ (1998) 50 Stan L Rev 1551.

20 Korobkin (n 19) 56.

21 Casey and Niblett (n 15) 1407.

22 FSMA 2000, s 3B(1)(b).

23 Casey and Niblett (n 15). H-B Schaefer, ‘Legal Rules and Standards’ in CK Rowley and F Schneider (eds) *The Encyclopedia of Public Choice* (Springer 2004) 347, 347–8; Korobkin (n 19) 46, 56 (arguing that the cost of using standards is higher because more cases will be litigated, and that self-serving bias increases the likelihood of litigation).

24 Casey and Niblett (n 15) 1408.

25 Micheler and Whaley (n 16) 364–5 (asserting that the cost of switching to the relevant technology is enormous but, once done, there will be massive savings collectively).

26 James Proudman, ‘Supervisor-Centred Automation—the Role of Human-Centred Automation in Judgment-Centred Prudential Supervision’ speech given at the Bank of England, CEPR and Imperial College Conference on the ‘Impact of AI and Machine Learning on the UK Economy’, 27 April 2020 <www.bankofengland.co.uk/speech/2020/james-proudman-supervisor-centred-automation-speech> accessed 20 August 2021; Mark Carney, ‘New Economy, New Finance, New Bank’ (21 June 2018), (speech on the ongoing work of the Bank to explore how new technologies could improve the compliance of the regulated industry and streamline regulatory processes while enhancing the ability of the Bank of England to analyse data) <www.bankofengland.co.uk/speech/2018/mark-carney-speech-at-the-lord-mayors-bankers-and-merchants-dinner-mansion-house> accessed 20 August 2021.

27 Casey and Niblett (n 15).

28 ibid 1416–17.

29 Casey and Niblett (n 15) 1411–12.

30 ibid 1404.

31 In a similar way that a ‘smart’ red-light camera can impose a fine by deducting the relevant amount of money from the bank account of a car driver. On the constitutional implications of the automation of enforcement in the United States, see JO Christensen, ‘Note, Wrong on Red: The Constitutional Case against Red-Light Cameras’ (2010) 32 Washington University Journal of Law and Policy 443, 446 (arguing that the use of these cameras violates the defendant’s right of due process).

32 Suitability requirements mainly apply to the provision of financial advice, investment management and the management of assets of certain pension schemes. FCA Handbook of Rules and Guidance, Conduct of Business Sourcebook (COBS), ch 9 <www.handbook.fca.org.uk/handbook/COBS/9/?view=chapter> accessed 12 August 2021.

33 A notable example is the ISDA Common Domain Model (CDM)—a logical model to express reporting rules for EMIR and MiFIR—which was tested in the DRR pilot. <www.isda.org/2019/10/14/isda-common-domain-model/> accessed 12 August 2021.

34 For a classic definition of machine learning, see T Mitchell, *Machine Learning* (McGraw-Hill Education 1997) 2. See also JD Lohr, WJ Maxwell and P Watts, ‘Legal Practitioners’ Approach to Regulating AI Risks’ in M Lodge and K Yeung (n 12) 224, 225 (providing examples of the multiple applications of machine learning).

35 The National Cyber Security Centre defines Distributed Ledger Technology as ‘a data storage mechanism in which data is stored at multiple locations on a shared network.’ It can be permissioned or permissionless, depending on the presence or absence of a central authority, which may be private or public. National Cyber Security Centre, *Distributed Ledger Technology—The nature and applications of Distributed Ledger Technology* (White Paper, 20 April 2021). <www.ncsc.gov.uk/whitepaper/distributed-ledger-technology#section_2> accessed 20 August 2021. Big Data describes an extremely vast set of accessible data (eg the Internet of Things). Big Compute refers to a wide range of tools and approaches to run large-scale applications for business, science and engineering performing complex modelling, simulations, etc. Cloud computing is an example of Big Compute. The Internet of Things connects machines and enables them to transfer data over the network autonomously—that is, without human-to-human or human-to-machine interaction. See further ‘FinTech Jargon Buster’, Practical Law UK Practice Note w-010-983; for a sceptical account on the allegedly positive impact of Big Data on Machine Learning, see DH Wolpert and WG Macready, ‘No Free Lunch Theorems for Optimization’ (1997) 1 IEEE Transactions on Evolutionary Computation 67.

36 XBLR is an example of semantic technology which provides a framework for exchanging business information <https://en.wikipedia.org/wiki/XBRL>, <https://en.wikipedia.org/wiki/XML> accessed 12 February 2020. For a critical examination of current initiatives to expand the use of AI and other technologies in the legal profession in the United States, see F Pasquale, ‘A Rule of Persons, not Machines: The Limits of Legal Automation’ (2019) 87 Geo Wash L Rev 1, 17–43.

37 Y LeCun, Y. Bengio and G Hinton, ‘Deep Learning’ (2015) 521 Nature 436.

38 For an overview of notable examples, see (Bank of England Transforming Data Collection Plan) (n 3) para 5.3; ISDA, ‘CDM Factsheet’ (2019) <www.isda.org/a/z8AEE/ISDA-CDM-Factsheet.pdf> accessed 20 August 2021.

39 Recent literature has moved beyond the traditional binary choice between rules and standards. C Ford, *Innovation and the State: Finance, Regulation and Justice* (CUP 2017) 12; Schauer, ‘The Convergence of Rules and Standards’ (n 18).

40 The letter ‘R’ in ‘COBS 2.1.1 (1) R’ earmarks COBS 2.1.1 as a ‘rule’. The word ‘Rules’ in the title ‘Fundamental Rules’ of the relevant part of the PRA Rulebook serves the same purpose. The legal status of the PRIN and the FR as ‘rules’ is also evident in the FCA and PRA Glossaries, both of which refer to s 417 (1) of the FSMA 2000, as the latter defines the term ‘rule’ broadly to include principles. <www.handbook.fca.org.uk/handbook/glossary/G1036.html?starts-with=R>, <www.prarulebook.co.uk/rulebook/Content/Chapter/211144/07-06-2022> accessed 1 July 2022.

41 Examples of high-level principles common to both the PRA and the FCA include the principles of integrity, of due care skill and diligence, and of financial prudence. See respectively PRIN 2.1.1 R <www.handbook.fca.org.uk/handbook/PRIN/2/?view=chapter> and FR 2.1 <www.prarulebook.co.uk/rulebook/Content/Chapter/211141/30-11-2015> accessed 1 July 2022.

42
*British Bankers Association, R (on the application of) v Financial Services Authority and another* [2011] EWHC 999 (Admin), para [170]. For a more recent application, see *Berkeley Burke SIPP Administration Ltd v Financial Ombudsman Service Ltd* [2018] EWHC 2878 (Admin).

43 J Black, ‘Paradoxes and Failures: New Governance Techniques and the Financial Crisis’ (2012) 75 MLR 1037, 1042–4; J Black, ‘The Rise, Fall and Fate of Principles Based Regulation’ (2010) LSE Law, Society and Economy Working Papers 17/2010 <http://ssrn.com/abstract=1712862> accessed 25 July 2022. See further A Georgosouli, ‘Judgement-Led Regulation: Reflections on Data and Discretion’ (2013) 14 Journal of Banking Regulation 209 (on the reincarnation of principles-based regulation as ‘judgment-led’).

44 Software development involves coding and computer programming. Coding is the process of translating and writing codes from one language to another, while programming is the process of building an executable programme <https://en.wikipedia.org/wiki/Computer_programming> accessed12 February 2020. See further JA Kroll and others, ‘Accountable Algorithms’ (2017) 165 U Pa L Rev 633, 646 (describing ‘software code’ as ‘a rigid and exact description of itself: the code both describes and causes the computer’s behavior when it runs’).

45 M Zalnieriute, L Bennett Moses and G Williams, ‘The Rule of Law and Automation of Government Decision-Making’ (2019) 82 MLR 425, 432–3.

46 Currently, regulators use rules to instruct regulatees to submit data for reporting purposes. Regulatory technology bears the potential of replacing the existing ‘push model’ of reporting with a ‘pull model’ of reporting in which regulators pull data themselves. For present purposes, I am assuming that the ‘push model’ of reporting remains in place, namely one in which regulatees have a legal obligation to submit information in compliance with regulatory law. On the distinction between different models of reporting, see Bank of England Discussion Paper (n 2) 42–5 <www.bankofengland.co.uk/-/media/boe/files/paper/2020/transforming-data-collection-from-the-uk-financial-sector.pdf?la=en&hash=6E6132B4F7AF681CCB425B0171B4CF43D82E7779> accessed 1 July 2022.

47 For a discussion on the use of technology in the private sector for the prediction of legal outcomes, see notably DM Katz, ‘Quantitative Legal Prediction—or How I Learnt to Stop Worrying and Start Preparing for the Data-Driven Future of the Legal Services Industry’ (2013) Emory LJ 909, 914–15; A Porat and LJ Strahilevitz, ‘Personalising Default Rules and Disclosure with Big Data’ (2014) 112 Mich L Rev 1417, 1436. Having in place tailored regulation for a particular financial institution is, of course, appealing; however, the use of the relevant technology by the industry is problematic where the incentives of the industry do not align with those of the regulators. Micheler and Whaley (n 16) 363 and 366.

48 For a discussion of relevant difficulties in the context of digital reporting, see FCA, ‘Pilot Phase 1 Report’ (n 5) 10, 14.

49 Lohr and others (n 34) 231 (noting that ‘Data is the raw material of machine learning’); Wolpert and Macready (n 35) 67–82 (taking a more sceptical stance and arguing that mining a larger pool of historical data will not make machine outputs correct or meaningful).

50 Bank of England and FCA, ‘Machine Learning and the UK Financial Services’ (October 2019) 21–3 <www.bankofengland.co.uk/report/2019/machine-learning-in-uk-financial-services> accessed 21 August 2021 (drawing a distinction between three different types of data: ‘structured’, ‘semi-structured’ and ‘unstructured-data’).

51 Hart uses this example to explore the ‘open texture’ of the law. HLA Hart, *The Concept of Law* (OUP 1994) 128–9.

52 GEM Anscombe and R Rhees (eds), *Philosophical Investigations* (GEM Anscombe tr, Blackwell 1953) paras 65–6 (on family resemblance). M McGinn, *Routledge Philosophy Guidebook to Wittgenstein and the Philosophical Investigations* (Routledge 2003) 33–72.

53 Reports are submitted to the FCA, but the FCA shares the relevant data with the PRA. On mortgage reporting requirements, see ‘FCA and PRA Changes to Mortgage Reporting Requirements’ (September 2019) Policy Statement FCA-PS19/23 PRA-PS22/19 <www.fca.org.uk/publication/policy/ps19-23.pdf> accessed 12 February 2020. Another example that could be mentioned here is the sustainability and environmental, social and governance reporting rules found in 9.8.6(8) R of the Listing Rules of the FCA Handbook (<www.handbook.fca.org.uk/handbook/LR/9/8.html>; accessed 12 May 2023). Some of the requirements (eg greenhouse emissions) have clear quantitative metrics and, as a result, they would lend themselves to digital reporting. Others would not, because of their subjective and open-ended nature (eg the description of the resilience of the organisation’s strategy in relation to climate risk).

54 According to SUP 16.11.7 R, the submission of PSD001 data reports must further meet the specifications of SUP 16 Annex 21R. <www.handbook.fca.org.uk/form/sup/SUP_16_ann_21_20191028.pdf> accessed 20 August 2021.

55 SUP 16.11.7 R.

56 Schauer, *Playing by the Rules* (n 17) 142.

57 Micheler and Whaley (n 16) 354 (noting in relation to natural language processing that the relevant technology is not sophisticated enough to cope with social context and the linguistic nuance of regulatory law). See further Zamir and Teichman (n 19) 141, 152 (considering how context affects people’s heuristics, biases and ultimately decisions, including those relevant to rule following).

58 Bank of England Transforming Data Collection Plan (n 3) para 3.3 (describing how regulatees engage with financial regulators, industry associations, peers and other stakeholders in their attempt to interpret reporting requirements, hence highlighting the participatory and dialectical character of that process). The procedural aspects of human interpretation and their importance are discussed further in s 5 below.

59 In relation to private law transactions, lawyers are also accustomed to the use of open-ended legal language in their attempt to eliminate the need to draft fully contingent agreements. JM Skarloff, ‘Smart Contracts and the Cost of Inflexibility’ (2017) 166 U Pa L Rev 263; EP Schuster, ‘Cloud Crypto Land’ (21 November 2018) LSE Legal Studies Working Paper 17/2019, (2021) 84 MLR 24. (discussing the practical value and cost efficiency of open-ended legal language in the context of smart contracts) <https://ssrn.com/abstract=3476678> accessed 20 August 2021.

60 Hildebrandt (n 16) 25. See further s 5 below, where I consider this issue in detail.

61 Here I draw on the mathematical concept of information of Claude E Shannon. See CE Shannon, ‘A Mathematical Theory of Communication’ (1948) 27 Bell System Technical Journal 623. For an insightful discussion of how maths is used to compress, process and transmit information (eg information about law) and further reference to literature on the mathematical theory of information and cybernetics, see Hildebrandt (n 16) 15–21.

62 Hildebrandt (n 16) 20.

63 J Burrell, ‘How the Machine “Thinks”: Understanding Opacity in Machine Learning Algorithms’ (2016) 3(1) Big Data and Society 12; Wolpert and Macready (n 35) 67–82; Zalnieriute and others (n 45) 425; Kroll and others (n 44), 633; FCA, ‘Pilot Phase 1 Report’ (n 5) 12.

64 M Oswald, ‘Algorithm-Assisted Decision-Making in the Public Sector: Framing the Issues Using Administrative Law Rules Governing Discretionary Powers’ (2018) 376(2128) Philosophical Transactions Royal Society 1, 7 <https://royalsocietypublishing.org/doi/abs/10.1098/rsta.2017.0359> accessed 25 August 2020.

65 CR Sunstein, ‘On Artificial Intelligence and Legal Reasoning’ (2004) 8 University of Chicago Law School Roundtable 29, 31–2; B Sheppard, ‘Incomplete Innovation and the Premature Disruption of Legal Services’ (2015) Michigan State Law Review 1787, 1870. On the nature of legal reasoning, see N McCormick, *Legal Reasoning and Legal Theory* (OUP 1978).

66 Here, I am only outlining a series of tasks that interpreters typically carry out. I am not concerned with the past decisions as formally binding precedent, which is a different matter. For a general discussion, see G Lamond, ‘Precedent and Analogy in Legal Reasoning’ *The Stanford Encyclopedia of Philosophy* (Spring 2016) <https://plato.stanford.edu/entries/legal-reas-prec/ accessed> 20 August 2021.

67 R Dworkin, *Law’s Empire* (Hart Publishing 1998) 65.

68 ibid 67–8.

69 Here I draw on the work of Ludwig Wittgenstein on ‘language games’ as aspects of a shared ‘form of life’. Anscombe and Rhees (n 52) paras 23, 203, 241; McGinn (n 52) 44, 55.

70 Georgosouli, ‘Regulatory Interpretation’ (n 10).

71 For a detailed analysis of different methods of machine learning, see G James and others, *An Introduction to Statistical Learning* (Springer 2017); I Goodfellow, Y Bengion and A Gourville, *Deep Learning* (MIT Press 2016). The complex calculations underpinning machine learning is known as algorithmic opacity. J Cobbe, ‘Administrative Law and the Machines of Government: Judicial Review of Automated Public Sector Decision Making’ (2019) 39 LS 635, 638–9.

72 Cobbe (n 71) 637–9.

73 A notable example is COBS 2.1.1 (1) R, known as ‘the client’s best interest rule’, according to which ‘A firm must act honestly, fairly and professionally in accordance with the best interests of its clients’ FCA Handbook of Rules and Guidance <www.handbook.fca.org.uk/handbook/COBS/2/1.html> accessed 12 February 2020. For a recent interpretation of the FCA’s client’s best interest rule, see *Quinn v IG Index Ltd* [2018] EWHC 2478 (Ch).

74 F Tomas, O Dodier and S Demarchi, ‘Computational Measures of Deceptive Language: Prospects and Issues’ (2022) 7 Frontiers in Communication 1, 10. <www.frontiersin.org/articles/10.3389/fcomm.2022.792378/full> accessed 20/5/22.

75 The Linguistic Inquiry and Word Count (LIWC) is a well-known example of computer-based textual analysis. See ML Newman and others, ‘Lying Words: Predicting Deception from Linguistic Styles’ (2003) 29 Personality and Social Psychology Bulletin 665, 666. For a critical overview of computational approaches to the detection of verbal deception, see Tomas and others (n 74).

76 Kroll and others (n 44) 653–6. On the inscrutability of various types of machine learning, see MH Jarrahi, ‘In the Age of the Smart Artificial Intelligence: AI’s Dual Capacities for Automating and Informating Work’ (2019) 36(4) Business Information Review 178, 182–3 (describing AI decision making as a ‘black-box’ compared to human decision making).

77 Oswald (n 64) 6 (using the provision of medical advice by way of example).

78 B Sheppard, ‘Warming Up to Inscrutability: How Technology Could Challenge Our Concept of Law’ (2018) 68 (suppl 1) UTLJ 36, 46–7.

79 ibid 46.

80 ibid.

81 For a similar observation focusing on regulators, see Lodge and Yeung (n 12) 23.

82 For instance, making rules almost always includes consultation with stakeholders, whereas applying rules (eg in order to assess compliance or enforce the law) requires regulatees to co-operate with the regulator and the regulator to make a transparent and *intra vires* decision. My conception of human interpretation as constructive deliberation is not meant to correspond strictly with either of these processes. Rather, it abstracts from their differences to bring attention to a feature that both share in common, namely that of constructive deliberations through which regulatory law is interpreted in order to be brought into life.

83 On current concerns about automation and how it would undermine rule-of-law principles if algorithms were to substitute human judgment and discretion, see notably Hildebrandt (n 16) 1; Pasquale (n 36) 17–43. For a proceduralist account of the rule of law, see J Waldron, ‘The Concept and the Rule of Law’ (2008) 43 Ga L Rev 1. For a discussion of formal and substantive accounts of the rule of law, see P Craig, ‘Formal and Substantive Conceptions of the Rule of Law [1997] PL 467.

84 The participation of leading industry players in the FCA pilot of DRR and other flagship initiatives suggests that there is a constant demand for automation from some part of the industry. At the same time, it raises concerns of regulatory capture. For a critical overview of the phenomenon of regulatory capture, see D Carpenter and DA Moss (eds), *Preventing Regulatory Capture: Special Interest Influence and How to Limit It* (CUP 2014).

85 J Raz, *The Morality of Freedom* (Clarendon Press 1988) 53 (providing a classic exposition of this view).

86 Substantive accounts of legitimacy differ from proceduralist accounts in that they assess legitimacy by focusing on the content of the decisions that are communicated as instructions, orders or commands. By contrast, proceduralist accounts focus on procedural aspects of decision making. Hybrid theories of legitimacy combine insights of both accounts. S Hershovitz, ‘Legitimacy, Democracy and Razian Authority’ (2003) 9 Legal Theory 201, 212.

87 S Hershovitz, ‘The Role of Authority’ (2011) 11(7) Philosopher’s Imprint 11; ibid 210 (noting that the division between *rulers* and *subjects* is not to be exaggerated given the prominence of (quasi-) decentred schemes of decision making and governance).

88 Other noteworthy examples are the relationship between doctors and patients under their care and the relationship between athletes and their fitness instructors.

89 Hershovitz, ‘Legitimacy, Democracy and Razian Authority’ (n 86) 212–13.

90 ibid.

91 ibid 214.

92 ibid 213–14.

93 On the distinction between practical authority and other forms of authority, see S Darwall, *Morality, Authority and Law* (OUP 2013) 135.

94 For a classic account of legitimacy in regulatory studies, see Baldwin and others (n 17) 26–31.

95 Here I am assuming that the authority of financial regulators is better accounted for according to a mixed model. David Estlund’s epistemic proceduralism is a prominent case in point as it attempts to strike a sensitive balance between epistemic and non-epistemic considerations. See D Estlund, *Democratic Authority: A Philosophical Framework* (Princeton UP 2009); for criticism, see E Anderson, ‘An Epistemic Defence of Democracy: David Estlund’s Democratic Authority’ (2008) 5 Episteme 129, 135 (arguing that the case against ‘epistocracy’ is first and foremost based on non-epistemic grounds and values). See further JM Laux, *Public Epistemic Authority: Normative Institutional Design for the EU* (Mohr Siebeck 2022) 4, 36.

96 The PRA Liquidity Monitoring Metric (LMM) tool is an algorithm published in Excel for the calculation of liquidity metrics for reporting purposes. At present, this tool is for guidance only and firms are not allowed to use it for the submission of regulatory returns as required by the rules. However, this may change in the future. The LMM figures amongst the Bank’s ‘use cases’, the upgrading of which it is hoped will lead to the delivery of ‘instructions as code’ for a more flexible reporting. PRA, ‘Supervisory Tools: Liquidity Tools’ <www.bankofengland.co.uk/prudential-regulation/publication/2013/supervisory-tools-liquidity-tools> accessed 19 August 2021; Bank of England Transforming Data Collection Plan (n 3) para 6.2.

97 Bank of England Transforming Data Collection Plan (n 3) para 4.3 (offering an insight into industry concerns).

98 D Ariely, *Predictably Irrational: The Hidden Forces that shape our Decisions* (Harper Collins 2009).

99 Lodge and Yeung (n 12) 31.

100 Georgosouli, ‘Judgement-Led Regulation’ (n 43).

101 On the historical evolution of financial regulation in the UK, see LCB Gower, ‘Big Bang and City Regulation’ (1988) 51 MLR 1 (describing the eventual mutation of self-regulation into statutory regulation); J Black, ‘Regulatory Styles and Supervisory Strategies’ in M Moloney E Ferran and J Payne (eds), *The Oxford Handbook of Financial Regulation* (OUP 2015) 217, 219–21.

102 M Lodge and A Mennicken, ‘Reflecting on Public Service Regulation by Algorithm’ in Lodge and Yeung (n 12) 178, 180 (viewing algorithmic regulation as an extension of existing regulatory systems but of different nature).

103 Pasquale (n 36) 3.

104 The choice of legal form and ultimately the design of financial rulebooks is a matter of balancing a series of competing policy considerations. For a classic discussion, see Diver (n 19) 73–4.

105 On the statutory objectives of the FCA, see FSMA 2000, s 1B(2) (defining the FCA’s ‘strategic objective’ as ensuring that ‘markets function well’). On the FCA’s ‘operational objectives’, see FSMA 2000, ss 1C (consumer protection objective), 1D (market integrity objective) and 1E (competition objective). On the Bank of England’s statutory objectives when acting as the PRA, see FSMA 2000, ss 2B (defining the authority’s ‘general objective’ as ‘promoting the safety and soundness of PRA-authorised persons’), 2C (insurance objective) and 2H (on the authority’s secondary competition objective).

106 Oswald (n 64) 16–17; M Nielsen, ‘How Computers Are Changing the Way We Explain the World’ *Wired Magazine* (August 2015) 8 <http://www.wired.com/2015/08/computers-changing-way-explain-world/?> accessed 20 February 2020.

107 Zamir and Teichman (n 19) 393–9; CR Sunstein, ‘Nudges.gov: Behaviorally Informed Regulation’ in E Zamir and D Teichman (eds), *The Oxford Handbook of Behavioral Economics and the Law* (OUP 2014) 719, 721–5; J Rachlinski and C Farina, ‘Cognitive Psychology and Optimal Government Design’ (2002) 87 Cornell L Rev 549; RB Korobkin and TS Ulen, ‘Law and Behavioral Science: Removing the Rationality Assumption from Law and Economics’ (2000) 88 Cal L Rev 1051; N Rangone, ‘Making Law Effective: Behavioural Insights into Compliance’ (2018) 9 EJRR 483, 484.

108 Creative compliance may stand at odds with the imperatives of judgment-led supervision, but it is a rational approach to follow from a cost-efficiency point of view.

109 On the treatment of improper delegation by UK administrative courts, see notably *Ellis v National Dock Labour Board* [1953] 2 QB 18; *H Lavender & Sons v Minister of Housing and Local Government* [1970] 1 WLR 1231 (taking advice from others does not amount to improper delegation as long as public officials did not have the decision dictated to them); *R v Home Secretary Co Ltd v Minister of Technology* [1971] AC 610, 625. On the treatment of irrelevant considerations, see *R v St Pancras Vestry* (1890) 24 QBD 371, 375; *R v Home Secretary ex p Venables* [1998] AC 407.

110 In the United States, this shift of perceptions on due process seems to be happening already in relation to criminal law proceedings. See notably *State v Loomis*, 881 NW2d 749 (Wis 2016), in which the sentencing judges referred to an algorithmic risk assessment tool without disclosing the methodology of the risk assessment that was embedded in the tool. The Supreme Court of Wisconsin found no violation of due process. It concluded that judges made an independent decision because they referred to the algorithmic risk assessment tool only as a source of information. Critics argue that the secrecy surrounding the modelling of the tool hampers any meaningful scrutiny of the reasoning of the judges. For instance, it is not possible to tell what data points were selected as relevant for the assessment of risk. See ‘Criminal Law—Sentencing Guidelines—Wisconsin Supreme Court Requires Warning before Use of Algorithmic Risk Assessments in Sentencing—State v Loomis 881 NW2d 749 (Wis 2016)’ (2017) 130 Harv L Rev 1530; F Pasquale, ‘Secret Algorithms Threaten the Rule of Law’ *MIT Technology Law* (1 June 2017) <www.technologyreview.com/2017/06/01/151447/secret-algorithms-threaten-the-rule-of-law/> accessed 1 October 2020. Similar concerns have been voiced in the UK in relation to the so-called Harm Assessment Risk Tool (HART), which is deployed by police to support their decisions notwithstanding the openness of the British police as regards features of the HART’s modelling. For an insightful analysis, see notably Oswald (n 64) 7.

111 Contrast here with the non-pejorative and purely technical use of the term ‘bias’ in probability, statistics and machine learning and social sciences as described by T Scantamburlo, A Charlesworth and N Christianini, ‘Machine Decisions and Human Consequences’ in Lodge and Yeung (n 12) 49, 57–8.

112 ibid 75–6. On the degradation of cognitive skills, see further K Volz and others, ‘An Evaluation of Cognitive Skill Degradation in Information Automation’ (2016) 60 Proceedings of Human Factors and Ergonomics Society Annual Meeting 191.

113 Here I am neither making a strong claim about the universality of empathy nor, indeed, claiming that there is a necessary connection between empathy and altruism. My claim is far more modest in that it holds that empathy can serve as a useful psychological condition for the delivery of certain desirable goals. In relation to the delivery of consumer protection, empathy can motivate financial advisors to, for example, have the interests of their clients at heart. Similarly, in relation to the preservation of financial stability, empathy can be instrumental in inducing systemically significant financial firms to consider the enormity of societal costs of reckless securities trading practices. For a critical examination of the role of empathy in morality, see notably HL Maibom (ed), *Empathy and Morality* (OUP 2014).

114 The contrary phenomenon of algorithmic aversion is, of course, plausible. Here I am focusing on automation bias only because I am interested in the attenuation of the moral aptitude of human rulebook users as a result of automation. SV Shiffrin, ‘Inducing Moral Deliberation: On the Occasional Virtues of Fog’ (2010) 123 Harv L Rev 1214, 1222, 1244. For a more general discussion, see L Alexander and E Sherwin, *The Rule of Rules: Morality, Rules and the Dilemmas of Law* (Duke UP 2001). On algorithmic aversion, see N Castelo, MW Bos and DR Lehmann, ‘Task-Dependent Algorithm Aversion’ (2019) 56 Journal of Marketing Research 809; BJ Dietvorst, JP Simmons and C Massey, ‘Algorithmic Aversion: People Erroneously Avoid Algorithms after Seeing Them Err’ (2015) 144 Journal of Experimental Psychology: General 114.

115 Oswald (n 64) 7.

116 Despite its importance, I am not concerned here with the question of cost efficiency on purely methodological grounds, as the issue does not arise unless and until it can be shown that it is desirable and, in principle, feasible to design the recommended rulebooks.

117 The proposed financial rulebooks could also help put an end to the worsening of the incentives problem in judgment-led supervision, especially if combined with a series of other measures. Examples include training to cultivate an aptitude for informed exercise of discretion, investing on cross-disciplinary synergies to improve the reviewability of metarules, and governance controls over how the industry makes use of software experts and other external professionals.

118 Hildebrandt (n 16) 30; Kroll and others (n 44) 699–705.

119 K Yeung, ‘Regulating Blockchain: The Emerging Battle for Supremacy between the Code of Law and Code as Law’ (2019) 82 MLR 207, 220–4.

120 HM Treasury, ‘Financial Services: The Edinburgh Reforms’ (9 December 2022). <www.gov.uk/government/collections/financial-services-the-edinburgh-reforms> accessed 30 April 2023 (announcing a set of reforms and emphasing the role of technological innovation in driving growth and competitiveness in financial services). E Ferran, ‘International Competitiveness and Financial Regulators’ Mandates: Coming Around Again’ (Faculty of Law, University of Cambridge 2022) Legal Studies Research Paper Series Paper No 6/2022 <https://papers.ssrn.com/sol3/papers.cfm?abstract_id=4144752> accessed 30 April 2023.

